# Distribution and drift dispersal dynamics of a caddisfly grazer in response to resource abundance and its ontogeny

**DOI:** 10.1098/rsos.160732

**Published:** 2017-01-25

**Authors:** Izumi Katano, Hiromune Mitsuhashi, Hideyuki Doi, Yu Isobe, Tadashi Oishi

**Affiliations:** 1 Faculty or Science, Nara Women's University, Kitauoya nishimachi, Nara 630-8506, Japan; 2 Museum of Nature and Human Activities, Yayoigaoka 6, Sanda 669-1546, Japan; 3 Graduate School of Simulation Studies, University of Hyogo, 7-1-28 Minatojima-minamimachi, Chuo-ku, Kobe 650-0047, Japan; 4 Nara Bunka Women's College, Higashinaka 127, Yamatotakada 635-8530, Japan; 5 G&L Kyousei Institute, 4-33 Yanagi, Yamatokoriyama 639-1134, Japan

**Keywords:** herbivore, algae and grazer interaction, food web, ontogenetic niche shift

## Abstract

Stream grazers have a major impact on food web structure and the productivity of stream ecosystems; however, studies on the longitudinal (upstream versus downstream) and temporal changes in their drift dynamics and resulting distributions remain limited. Here, we investigated the longitudinal and temporal distributions and drift propensity of a trichopteran grazer, the caddisfly, *Micrasema quadriloba*, during its life cycle in a Japanese stream. The distribution of larvae significantly shifted downstream during the fifth instar larval stage during late winter; with periphyton abundance (i.e. their food source) showing similar shifts downstream. Therefore, our results show that the drift dispersal the caddisfly occurs in response to decline in available food resources (i.e. food-resource scarcity) and an increase in food requirements by growing individuals. Furthermore, our results show that this observed longitudinal shift in larval distribution varies through their life cycle, because the drift dispersal of fifth instar larvae was greater than that of immature larvae. The correlation between periphyton abundance and drift propensity of fourth instar larvae was not statistically significant, whereas that of fifth instar larvae was significantly negative. In conclusion, we detected an ontogenetic shift in drift propensity, which might explain the longitudinal and temporal distributions of this species.

## 1. Introduction

One of the unique characteristics of stream ecosystems is the unidirectional transport of materials, forming the basis of the river continuum concept [1]. Because this transport of materials includes organisms [2,3], the drift of benthic invertebrates has received considerable attention from stream ecologists [3–5]. Further, the dispersal of species is essential to maintain gene flow and genetic diversity of invertebrate populations, as well as for the colonization and re-colonization of habitats [6,7]. Ephemeroptera, Diptera, Plecoptera and Trichoptera are common drift taxa, with baetid nymphs (Ephemeroptera) constituting the major component [8–11].

Drift dispersal by organisms is be categorized into three types: catastrophic, constant and behavioural [12], with behavioural drifts being the most prevalent. The behavioural drift of invertebrates is mainly driven by the presence of predators [11,13–16] and food availability [17–19]. Indeed, drift is a function of *per capita* food demand, rather than interference or the density of individuals [19,20]. Consequently, drift results from increased searching activity for resources when available resources are low [21]. Kohler [22] hypothesized that individuals tend to aggregate in habitats where habitat quality (e.g. food resource abundance) is high. Experiments testing this hypothesis [23–26] found that drift and habitat-use behaviours are induced by resource availability. However, descriptions of longitudinal and temporal changes in drift and habitat quality remain limited in natural conditions, except for baetid nymphs [18,20]. Trichopteran grazers have a major impact on periphyton abundance and their communities, which, in turn, affects the whole food web of stream ecosystems [27–30]. However, longitudinal and temporal changes in the drift and subsequent distribution of these grazers require investigation in the natural environment.

Ontogenetic changes in dispersal behaviour and species distribution have been recorded for many organisms, resulting in ecologists recognizing their importance in the life cycles of certain species, habitat use and population dynamics [31]. In fact, many studies suggest that ontogenetic shifts contribute to the structure and dynamics of populations by adjusting the requirements of a species to the spatial and temporal dynamics of environmental and resource conditions [32,33]. However, studies on drift behaviour of stream invertebrates have not considered ontogenetic effects, even though ontogenetic changes in drift behaviour probably occur.

In this study, we investigated the longitudinal and temporal distribution and drift dispersal of a trichopteran grazer species, *Micrasema quadriloba*, in a Japanese stream in relation to the availability of their food resource (periphyton) and the increase in resource requirements of maturing individuals in natural streams. This objective was achieved by comparing the longitudinal and temporal distributions of *M. quadriloba* larvae and periphyton abundance each month throughout the life cycle of the larvae. In parallel, we evaluated ontogenetic shift in drift behaviour of successive instars with increasing food requirements. This objective was achieved by comparing the drift propensities of the fourth and fifth instar stages in relation to resource levels.

## 2. Material and methods

### 2.1. Study organism

Our study species was a case-bearing caddisfly *M. quadriloba* Martynov (Brachycentridae). The larvae are low-mobility grazers that are widely distributed in the mountain streams of central Honshu, Japan [29]. This species is univoltine, hatching in June, pupating in March of the following year, and emerging in May [34]. Adult females fly upstream of the emergence sites, where they lay their eggs [35]. The eggs hatch in early summer (mainly June). The larvae graze on periphyton, which grow on the substrata [36].

### 2.2. Study area

**Figure 1.**
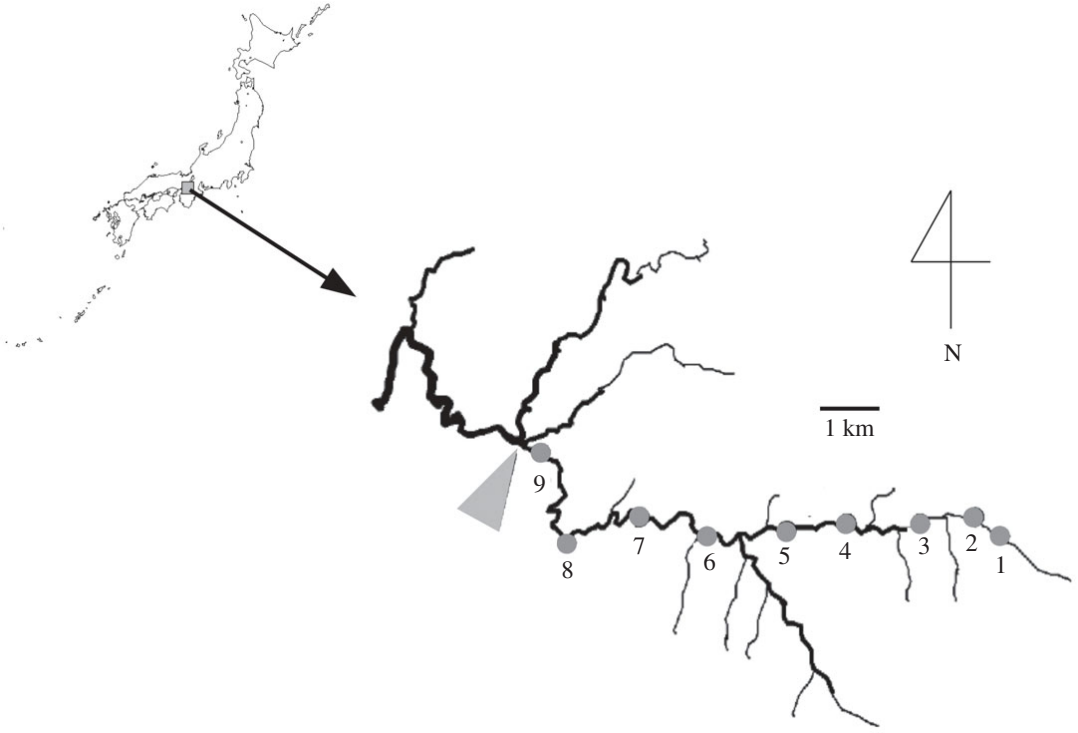
Map of the nine sampling stations in the Shigo-gawa Stream, Japan. The large arrow shows the station where the tributary flows into the stream.

The survey was conducted in the Shigo-gawa Stream (stream width: 2–18 m, mean gradient: 2.2%), which is a second-order mountain stream in Higashi-yoshino, Japan (34°22.7′ N, 136°1.0′ E, figure 1). Nine survey stations were established along an 11.7 km stretch, which was 0.4–12.4 km upstream from the starting station of the third-order stream (figure 1). The substrates of the streambed were mainly boulders and cobbles [29,36]. The riparian zone of this stretch was dominated by artificial forests of Japanese cedar, *Cryptomeria japonica*.

### 2.3. Investigation of longitudinal and temporal distributions

We conducted the field surveys every month from November 2001 to February 2003 at the nine stations (figure 1). Sampling dates were 15 November and 17 December 2001; 23 January, 22 February, 26 March, 25 April, 16 May, 27 June, 27 July, 28 August, 30 September, 29 October, 29 November and 27 December 2002; and 27 January and 27 February 2003. On each sampling date, water temperature and electrical conductivity (EC) were measured at each station at midday using a thermometer and an EC metre (CM-14P, TOA Electronics Ltd., Tokyo, Japan). Precipitation data for the region were obtained from the nearest meteorological observatory to the nine stations, Nara Local Meteorological Observatory (34°29.3′ N, 135°55.9′ E).

To measure the benthic density of *M. quadriloba* larvae, 20 points were established with the same intervals on two lines across the stream width (1 m apart) in a riffle at each station. At each point, the maximum number of *M. quadriloba* in 25 cm^2^ (5 × 5 cm quadrat) on a cobble was counted and collected. The collected larvae were immediately preserved in 5% buffered formalin solution, and species identification was confirmed with a binocular microscope in the laboratory.

To identify the amount of periphyton at each station, the amount of chlorophyll *a* was measured on 10 cobbles along a downstream line across the stream where the number of *M. quadriloba* individuals was counted. The periphyton on a cobble were wiped using an acrylic fibre cloth in a circle of 3 cm in diameter according to the acrylic fibre sampler method [37]. The fibre clothes were placed into vials containing 10 ml of 99.5% ethanol. After preservation in the dark at 4°C for 24 h, the extracted pigment was measured using a spectrophotometer (Model MPS-2000, Shimadzu, Tokyo, Japan). Chlorophyll *a* was determined according to SCOR-UNESCO [38].

### 2.4. Drift and benthic sampling and environmental factors

Drifting insects were collected on 19 and 20 December 2002 and on 7 and 8 February 2003, using a drift-net (mesh of 300 µm, mouth opening of 15 × 15 cm, net length of 0.5 m) at the riffles of four stations (stations 2, 4, 6 and 8; figure 1). At each sampling station, four drift nets were placed across the stream for 1 h from 13.00 to 14.00 and from 14.00 to 15.00. Therefore, eight replicates of drift sampling at each station were collected. Current velocities, water temperature and electric conductivity at the net openings were measured using a current metre (CR-11, Cosmo-Riken, Osaka, Japan), a thermometer and an EC metre (CM-14P, TOA Electronics Ltd.), respectively.

To measure the benthic density of *M. quadriloba* larvae and to examine the species composition of invertebrates, the four benthic samples were collected using a server-net sampler (25 × 25 cm quadrat, mesh size: 0.5 mm) at 1 m upstream of each station on the same days as the drift sampling. Both the drift and benthic samples were immediately preserved in 5% buffered formalin solution, and were subsequently examined with a binocular microscope in the laboratory. The number of drift and benthic *M. quadriloba* larval individuals was used to calculate the drift and benthic density, respectively. Drift density was expressed as the number of *M. quadriloba* larvae in 100 m^3^ water [13]. Benthic density was expressed as the number of *M. quadriloba* larvae per square metre. We subsequently estimated the drift propensity of larvae (unit: 1/100 m) at each sampling station and period, by dividing the mean drift density with mean benthic density [14]. An increase in the drift propensity index means reflects an increase in the number of drift individuals compared with benthic individuals. The calculated drift propensities in December and February reflected the drift propensity of fourth and fifth instars, respectively, because these larvae stages dominated the samples at these respective time points (see Results section).

To quantify the amount of periphyton at each station, chlorophyll *a* was measured in a cobble per quadrat at the same time as the benthic sampling (*n* = 4). Chlorophyll *a* was determined according to the method described above.

### 2.5. Statistical analysis

Temporal changes in longitudinal distribution were analysed using a general additive model (GAM) with the loss smooth function and Gaussian error distribution. A generalized linear model (GLM) with Gaussian error distribution and log-link function was used to analyse the correlation between the mean benthic density of larvae and the mean amount of periphyton on all dates, except from March to May 2003. We also performed a GLM (log-link function) for the relationships between the drift propensity and periphyton abundance at each instar stage. The normality of values was tested with the Shapiro–Wilk's normality test (*α* = 0.05). For all statistical tests, log_10_ (*x* + 1) transformations for exact values were made to standardize variance and improve normality. All statistical and graphical analyses were performed using R v. 3.2.2 [39].

## 3. Results

### 3.1. Environmental conditions and longitudinal distribution of *Micrasema quadriloba* larvae

Water temperature gradually increased downstream on each given sampling date, and was the highest in July and the lowest in January (electronic supplementary material, figure S1). By contrast, EC was highest at station 4 throughout the entire field survey period (electronic supplementary material, figure S1). Precipitation was highest in July and August 2002 and January 2003 (electronic supplementary material, figure S1). Disturbance by floods was likely, although the discharge of the stream was not measured.

**Figure 2.**
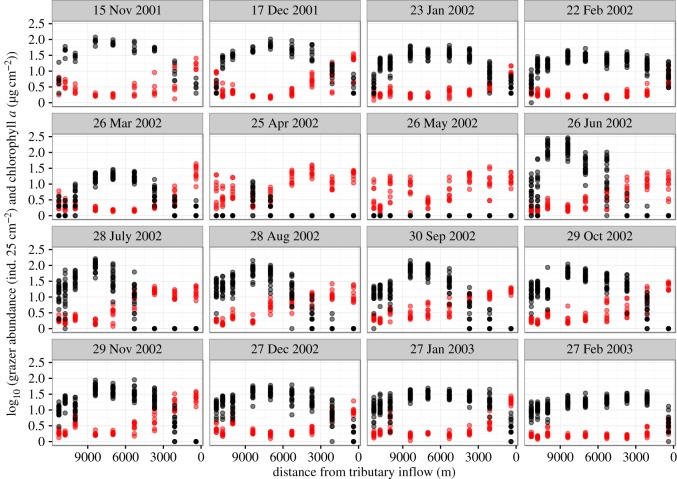
Distribution of *Micrasema quadriloba* larvae (black circle) and periphytic chlorophyll *a* (Chl-*a*, red) in the stream from November 2000 (11/2000) to February 2003 (2/2003). The distance from the tributary inflow on the *x*-axis was ordered from upstream to downstream. Numbers of pupae and egg masses are not shown. Note: In March and April 2002, the larvae changed to pupae gradually. In May 2002, egg masses were found downstream.

Temporal and longitudinal changes in periphyton abundance (Chl-*a*) and the density of *M. quadriloba* larvae are shown in figure 2. *Micrasema quadriloba* pupated from March to May 2002 and hatched in June 2002. Subsequently, the first instar larvae appeared in the stream. In the other sampling months, *M. quadriloba* larvae were distributed along the entire stream section.

Periphyton abundance (Chl-*a*) tended to be low at the stations where the greatest number of *M. quadriloba* larvae were found, irrespective of month (figure 2). For example, when larvae occurred along the investigated stretch of the river (November 2001–February 2002 and June 2002–February 2003), periphyton abundance increased downstream. However, in spring, when *M. quadriloba* developed from larvae to pupae, adults and egg masses, the winter distribution pattern in periphyton disappeared.

**Figure 3.**
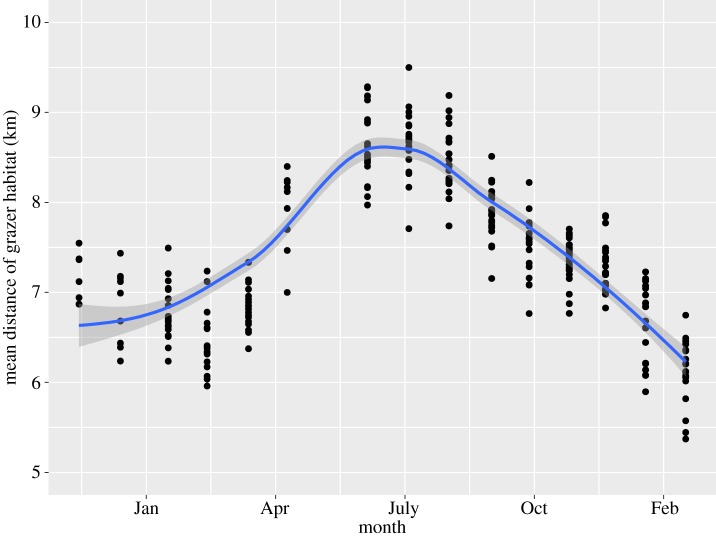
Mean longitudinal distributions of *Micrasema quadriloba* larvae in the sampling periods. In May 2002, the distance indicated longitudinal distribution of the egg masses of *M. quadriloba* instead of *M. quadriloba* larvae. The line indicates significant GAM regression with ±95% CI.

Temporal change in the mean distribution distance of larvae was unimodal pattern fitted by GAM (*F* = 151.0, *p* < 0.001, GCV = 0.135, *R*^2 ^= 0.832; figure 3). The longitudinal distribution of the larvae shifted from upstream to downstream after juveniles appeared upstream. A clear peak in distribution in the upper reaches was shown for the longitudinal distribution of larvae from June (hatching) to December (larvae were mainly fourth instar). From early winter to before pupation in February (all larvae were fifth instar, see below), larvae were evenly distributed along the stream (i.e. no peak), indicating that their distribution shifted downstream homogeneously.

**Figure 4.**
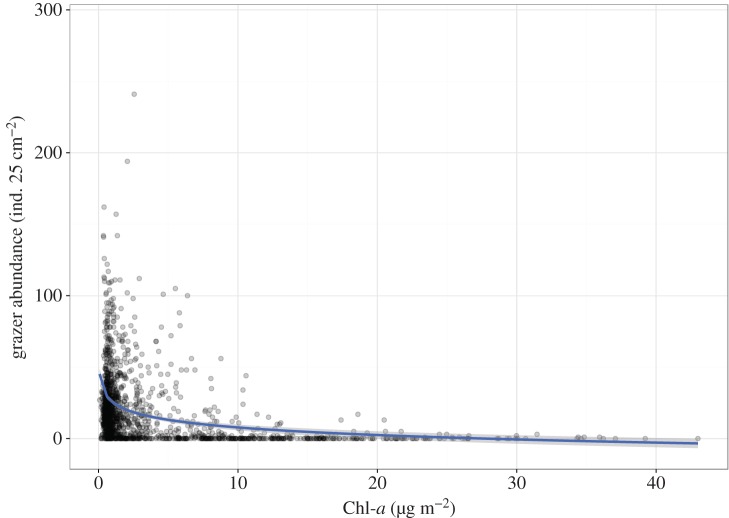
The relationship between the number of *Micrasema quadriloba* larval individuals and periphytic chlorophyll *a* (Chl-*a*), except for March, April and May 2002. The line indicates a significant linear regression (log function) with ± 95% CI.

During the sampling period, periphyton was abundant at station 4, except for where larvae were present. In total, the amount of periphyton significantly decreased with increasing numbers of *M. quadriloba* larvae (LM with log-link, *R*^2^ = 0.12, *p* < 0.001; figure 4).

### 3.2. Ontogenetic shift in drift propensity

**Figure 5.**
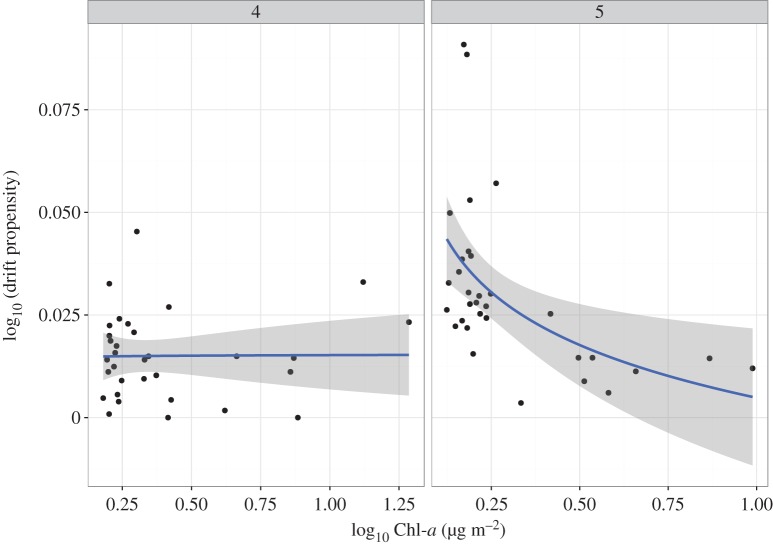
Relationship between the drift propensity of *Micrasema quadriloba* and periphytic chlorophyll *a* (Chl-a) for different instars; fourth instar larvae sampled on 19–20 December 2002 (4); fifth instar larvae sampled on 7–8 February 2003 (5). The line indicates a significant linear regression (log function) with ±95% CI.

The relationship between drift propensity and periphyton abundance (Chl-*a*) was not significant for fourth instar larvae (LM with log-link, *R*^2^ = 0.003, *p *= 0.753; figure 5), but was significantly negative for fifth instar larvae (*R*^2^ = 0.215, *p *< 0.001; figure 5). The slopes of the GLMs differed for fourth and fifth instar larvae, respectively (0.0022 and −0.045; figure 5).

At each station, the fourth and fifth instar larvae of *M. quadriloba* were dominant in December and February, respectively, representing more than 96% of all larvae. Mean benthic densities of *M. quadriloba* larvae and the mean amount of periphyton at each station ranged from 0.03 to 1.10 individuals cm^−2^ and from 0.50 to 6.95 µg Chl-*a* cm^−2^, respectively. *Micrasema quadriloba* larvae at each station represented 50–90% of all benthic invertebrate individuals.

## 4. Discussion

This study demonstrated that the longitudinal distribution of *M. quadriloba* clearly shifts downstream over the larval stages of their life cycle, before adults fly back upstream to lay eggs. We also showed that this shift in distribution might influence the longitudinal distribution of periphyton abundance, because of their feeding habits. The drift behaviour of certain stream invertebrates has been extensively reported for baetid nymphs [8,15,16]. However, few studies have demonstrated both drift dispersal and a downstream shift in their longitudinal distributions. Of note, periphyton abundance was depleted in the sampling stations where the greatest number of larvae was found, regardless of their growth stages.

The larvae of *M. quadriloba* were the dominant benthic invertebrate grazers during late winter, which significantly increased their drift propensity with the depletion of periphyton abundance (i.e. their food resource). This observation was with the findings of studies on baetid nymphs [18,20]. Kohler [22] suggested baetid nymphs abandon a habitat by drift at a certain threshold level of habitat quality. Nymphs are highly mobile, allowing them to migrate to alternative, resource-rich periphyton patches. By contrast, case-bearing *M. quadriloba* larvae are only able to crawl, resulting in their having comparatively lower mobility than baetid larvae. Thus, *M. quadriloba* larvae might continue to graze in the local neighbourhood, rather than move to other periphyton patches. Therefore, if *M. quadriloba* has an assumed threshold level to abandon a habitat, the level of resource abundance would be lower than that of baetid nymphs. This low threshold level might reflect the low drift propensity of fourth instar larvae. The maximum drift propensity of fifth instar larvae was approximately two times greater than that of fourth instar larvae, for which drift propensity was not correlated with periphyton abundance. This difference might be due to an increase in food requirements as they grow, which drives individuals to actively drift downstream. In winter, increasing food requirements of fifth instar larvae and depleted periphyton abundance might have exceeded the assumed threshold level, leading to their drifting.

Despite the scarcity of resources, the longitudinal distribution of *M. quadriloba* larvae did not shift downstream until late summer. The restricted longitudinal distribution might be passively affected by higher water temperatures in the downstream reaches (greater than 20°C). Water temperature is an important determinant of the longitudinal distribution of aquatic insects [11,40–41]. The absence of *M. quadriloba* larvae in the downstream reaches might be a result of ineffective dispersal (i.e. high mortality with high water temperatures) from June to late summer, because the larvae were found in mountain streams with higher currents and show a non-active period during the summer [42]. By contrast, from mid- to late winter, when *M. quadriloba* were fifth instar larvae, their longitudinal distribution shifted downstream, reflecting that also observed for blepharocerid larvae during winter [43]. In parallel, the sampling stations were depleted of periphyton, which were more abundant downstream. The food requirement of fifth instar larvae could not be met by low periphyton abundance in the local neighbourhood; thus, the larvae might alter their strategy and migrate to a new foraging habitat (i.e. increase the extent of their foraging area). Such movement might cause drift dispersal, resulting in the longitudinal distribution of fifth instars expanding downstream in winter.

In conclusion, the longitudinal and temporal distributions of the larvae of the caddisfly grazer, *M. quadriloba* might be regulated by their drift behaviours, which, in turn, might be driven by their resource requirements, changes in ontogeny and periphyton (resource) abundance. Resources and ontogeny might represent important factors in determining the longitudinal and temporal distribution of drifting stream invertebrates, with the drift dynamics of abundant grazers potentially shaping stream food webs, as well as ecosystem production and functioning through changes in the distribution in microorganisms (periphytic) involved in primary production.

## Supplementary Material

Fig. S1 Abiotic factors (water temperature, electric conductivity (EC), and precipitation) at each survey station in the Shigo-gawa stream during downstream surveys. Precipitation was observed at Nara Meteorological Observatory. Table S1. Environmental variables (mean ± 1 SD) of each sampling site. The values in parentheses are sample sizes.

## Data accessibility

Our data are deposited at the Dryad Digital Repository: http://dx.doi.org/10.5061/dryad.t58d9 [44].
