# Supplementary material for: Distribution and drift dispersal dynamics of a caddisfly grazer in response to resource abundance and its ontogeny
Source: R Soc Open Sci. 2017 Jan 25;4(1):160732. doi: 10.1098/rsos.160732 (PMC5319342; doi:10.1098/rsos.160732)
Supplement: Fig. S1 Abiotic factors (water temperature, electric conductivity (EC), and precipitation) at each survey station in the Shigo-gawa stream during downstream surveys. Precipitation was observed at Nara Meteorological Observatory. Table S1. Environmental variables (mean ± 1 SD) of each sampling site.  [file rsos160732supp1.docx]

Supplemental Materials

Fig. S1 Abiotic factors (water temperature, electric conductivity (EC), and precipitation) at each survey station in the Shigo-gawa stream during downstream surveys. Precipitation was observed at Nara Meteorological Observatory.

**Table S1.** Environmental variables (mean ± 1 SD) of each sampling site. The values in parentheses are sample sizes.
